# Identification of endoplasmic reticulum stress-related lncRNAs in lung adenocarcinoma by bioinformatics and experimental validation

**DOI:** 10.1080/07853890.2023.2251500

**Published:** 2023-08-29

**Authors:** Tong Xin, Yue Sun, Hongxue Meng, Ningzhi Zhang, Bo Peng, Xinxin Yang, Jing Hu, Mengru Cao

**Affiliations:** a The Fourth Department of Medical Oncology, Harbin Medical University Cancer Hospital, Harbin, China; b Department of Pathology, Harbin Medical University Cancer Hospital, Harbin, China; c Department of Thoracic Surgery, The Second Affiliated Hospital of Harbin Medical University, Harbin, China; d Precision Medicine Center, Harbin Medical University Cancer Hospital, Harbin, China

**Keywords:** Endoplasmic reticulum stress, prognosis, LncRNAs, lung adenocarcinoma

## Abstract

**Background:**

Endoplasmic reticulum stress (ERs) is an important cellular self-defence mechanism, which is closely related to tumorigenesis and development. However, the role of endoplasmic reticulum stress state in the development of lung adenocarcinoma (LUAD) has not been clarified.

**Methods:**

The lncRNAs associated with endoplasmic reticulum stress were identified by co-expression analysis in public databases, and by the least absolute shrinkage and selection operator (LASSO) regression and multivariate Cox regression modelling, we constructed a prognostic model based on endoplasmic reticulum stress-related lncRNAs (ERs-related lncRNAs), performed immune analysis, TME, TMB and clinical drug prediction for model-related risk scores, and performed correlation validation in public databases and at the human tissue level.

**Results:**

Five ERs-related lncRNAs were used to construct an ERs-related lncRNA signature (ERs-related LncSig), which can predict the prognosis of LUAD. Patients in the high-risk group had worse survival, and differences existed in immune cell infiltration, immune function, immune checkpoint analysis, tumour microenvironment (TME), tumour mutational burden (TMB), immunotherapy efficacy, and sensitivity to some commonly used chemotherapeutic agents between high and low risk groups.

**Conclusion:**

Our study demonstrated that ERs-related lncRNA signature can be used for the prognostic evaluation of LUAD patients and may provide new insights into clinical decision-making and personalised medicine for LUAD.

## 1. Introduction

In recent years, despite some advances in screening, diagnosis, and treatment of LUAD, its 5-year relative survival rate remains low[1]. Therefore, new molecular biomarkers to predict prognosis and treatment response in LUAD patients are urgently needed.

The endoplasmic reticulum (ER) is the main site of protein synthesis, processing, and transportation, as well as an important place for lipid metabolism and Ca^2+^ storage, when endogenous or exogenous stimuli cause a disturbance in the protein folding function of the ER, large amounts of unfolded or misfolded proteins accumulate in the ER lumen causes a series of subsequent reactions—a condition referred to as “ER stress”. When the accumulation of misfolded proteins exceeds a threshold, the unfolded protein response (UPR) will be stimulated to cope with this change. ER stress is a protective mechanism for ER homeostasis. During tumour growth, the uncontrolled proliferative capacity of tumour cells creates a deleterious microenvironment characterized by high metabolic demand, nutrient limitation, hypoxia, and acidosis. However, tumour cells can adapt to this hostile microenvironment by promoting ER stress and influencing their progression[2]. It has been shown that ER stress is important for tumorigenesis. For instance, Tripartite motif containing 25 (TRIM25) is the most important induced gene in response to ER stress, and ER stress leads to the increase of TRIM25, which promotes the development of liver cancer through the Kelch-like ECH-associated protein 1 (Keap1)/nuclear factor erythroid 2-related factor 2 (Nrf2) signalling pathway[3]. In bladder cancer cells, extracellular vehicles (EVS) can promote the proliferation of bladder cancer by stimulating UPR during ERs[4]. ER stress similarly plays an important role in LUAD, and studies have shown that certain compounds or drugs can act as tumour suppressors by acting on ER stress: Ficolin 3 (FCN3), a secreted lectin capable of activating the complement pathway, plays a role in suppressing LUAD by inducing ER stress[5]; β,β-Dimethylacrylshikonin (DMAS), an anti-cancer compound extracted from the roots of Lithospermum erythrorhizon, stimulated endoplasmic reticulum stress and mediated autophagy through the PERK-eIF2a-ATF4-CHOP and IRE1-TRAF2-JNK axes of the unfolded protein response in human lung adenocarcinoma cells[6]. Therefore, exploring the relationship between ER stress and LUAD tumour progression is necessary.

Long non-coding RNA (LncRNA) is defined as RNA longer than 200 nucleotides that are not translated into a functional protein, it plays an important role in many diseases including LUAD[7]. Many studies have proved that lncRNAs can affect tumour progression by regulating endoplasmic reticulum stress: in oesophageal and liver cancer, lncRNA MEG3 triggers apoptosis of tumour cells through the endoplasmic reticulum stress pathway[8, 9]; lncRNA CASC2 increases the stability of PERK mRNA, which triggers PERK/eIF2α/CHOP ER stress pathway and promotes radiosensitivity or apoptosis in non-small cell lung cancer (NSCLC)[10].

However, the mechanism of endoplasmic reticulum stress-related lncRNAs in LUAD has not been fully elucidated. In our study, we established an individualized signature comprising five ERs-related lncRNAs, which could be used to predict the prognosis of LUAD and distinguish LUAD patients’ responses to immunotherapy and chemotherapy drugs, providing new insights into the diagnosis and treatment.

## 2. Materials and methods

### 2.1. Data acquisition

The RNA transcriptome sequence data, mutation data, and clinical data were downloaded from the TCGA database (https://portal.gdc.cancer.gov/). The lncRNAs were distinguished by annotation files downloaded from the Ensembl database (https://asia.ensembl.org/) [11].

The ER stress-related genes were obtained from seven ER stress-related gene sets (GOBP_NEGATIVE_REGULATION_OF_RESPONSE_TO_ENDOPLASMIC_RETICULUM_STRESS,GOBP_POSITIVE_REGULATION_OF_RESPONSE_TO_ENDOPLASMIC_RETICULUM_STRESS, GOBP_POSITIVE_REGULATION_OF_TRANSLATION_IN_RESPONSE_TO_ENDOPLASMIC_RETICULUM_STRESS,GOBP_REGULATION_OF_RESPONSE_TO_ENDOPLASMIC_RETICULUM_STRESS,GOBP_REGULATION_OF_TRANSLATION_IN_RESPONSE_TO_ENDOPLASMIC_RETICULUM_STRESS,GOBP_REGULATION_OF_TRANSLATION_INITIATION_IN_RESPONSE_TO_ENDOPLASMIC_RETICULUM_STRESS, GOBP_RESPONSE_TO_ENDOPLASMIC_RETICULUM_STRESS) in Molecular Signature Database (https://gsea-msigdb.org/). In those sets, overlapped genes were removed and we acquired 256 genes for subsequent analysis.

### 2.2. Identification of ERs-related lncRNAs

41 potential prognostic ER stress-related genes were identified by univariate Cox hazard regression based on 256 ER stress-related genes. We selected 3435 differentially expressed lncRNAs and 41 potential prognostic ER stress-related genes performing Pearson correlation analysis, and identified 639 ERs-related lncRNAs. Differential lncRNAs were determined based on |log2 FC| > 1 and false discovery rate (FDR) < 0.05 thresholds.

### 2.3. Construction of the ERs-related lncRNA prognostic model

Based on the ERs-related lncRNAs with prognostic value, we used lasso analysis to further screen for prognostic factors, and then after multivariate Cox proportional hazards regression analysis, we screened out five ERs-related lncRNAs for the construction of a predictive signature. The risk score was calculated using the following risk formula:

$$Risk\;Score={\sum_{j\;=\;1}^{n}Coef\left(j\right)\;\;\text{*}\;\;i\left(j\right)}_{}^{}$$

Cases were divided into a high-risk group and a low-risk group by the median risk score for further studies.

### 2.4. Validation of the risk prognosis model

We contrasted the distribution of patients based on the results of unsupervised clustering of prognostic-related ERs-related lncRNAs and the results of risk grouping to verify the rationality of risk grouping. Then, the PCA analysis was performed to examine the clustering ability of the risk model.

The survival difference in two risk groups was calculated using the Kaplan-Meier method by R “survival” and “survminer” packages. Besides, univariate and multivariate Cox regression analysis were used to assess whether the risk score was independent of other clinicopathological parameters. We then constructed a nomogram with the R package "rms" and plotted a calibration curve to estimate the predictive performance of the nomogram.

### 2.5. Function enrichment analysis

The Gene Ontology (GO) analysis and Kyoto Encyclopaedia of Genes and Genomes (KEGG) analysis were identified in the R program. We also used the Gene Set Enrichment Analysis (GSEA, version 4.2.3)[12] and Gene Set Variation Analysis (GSVA, with “GSVA” R package)[13] to investigate the metabolic pathways involved in the different risk subgroups of LUAD stratified by ERs-related LncSig and the correlation between model genes and risk scores with these pathways.

### 2.6. Immune and tumor microenvironment analysis

We calculated the relative abundance of tumour-infiltrating immune cells (TIICs) in each sample with the “CIBERSORT” method and verified the difference between high- and low-risk groups with Wilcoxon test. For the accuracy of the results, we downloaded the information estimation data in TIMER2.0 (http://timer.cistrome.org) to verify the TIICs analysis of risk groups in other databases. Then a single-sample gene set enrichment analysis (ssGSEA) algorithm was used to analyze the relationship between the ERs-related LncSig and immune function. The Tumour Immune Dysfunction and Exclusion (TIDE) algorithm was used to calculate the response to immunotherapy[14]. Finally, we used R “estimate” package to calculate the immune score, stromal score, and ESTIMATE score for each LUAD sample.

### 2.7. Tumour mutation burden analysis

The R “maftools” package was used to visualize somatic mutation data in the mutation annotation format and calculate the TMB of LUAD patients.

### 2.8. Chemotherapy drug sensitivity and potential compounds

The “pRRophetic” package in R was used to analyze the relationship between risk grouping and the efficacy of chemotherapeutic agents that are commonly recommended for LUAD treatment. Next, we used the Connectivity map database (https://clue.io/) to analyze the potential compounds that are potentially associated with the risk scores.

### 2.9. Quantitative reverse transcriptase PCR (qRT-PCR)

Patient samples were collected at Harbin Medical University Cancer Hospital, including 7 pairs of LUAD and its paired normal tissues. The total RNA was extracted using the TRIzol reagent (Invitrogen, CA, United States). 1 µg total RNA and ReverTra Ace qPCR RT Master Mix (TOYOBO) were used for reverse transcriptase reaction. Then, 1 µl synthesized cDNA and SYBR Green Master Mix Kit (TOYOBO) were used for amplification. The levels of the five model lncRNAs were normalized with GAPDH as a reference, and the relative expression was calculated based on the comparative Ct (2 − ΔΔCt) method. The primer sequences used in this study were listed as follows

KTN1-AS1: Forward primer: 5′-CAACTTCTGGGTCCAGGCTA-3′
     Reverse primer: 5′-CTCAGGGCCTCTCTACATGG-3′
MIR223HG: Forward primer: 5′-CACCACTCCACTGACAGACT-3′
      Reverse primer: 5′-TGACCCTGGCAAAGTTGTTG-3′
CASC15: Forward primer: 5′-GCAACAGTATGGGCTCACAG-3′
     Reverse primer: 5′-GGCCCATGGATGGAGATGTA-3′
AC026356.1: Forward primer: 5′-TAAAAAGCATCTGGTTCC-3′
     Reverse primer: 5′-GCTCTCATTCTACATCGC-3′
AL606489.1: Forward primer: 5′-AGCCCCACCCATCCTTCC-3′
     Reverse primer: 5′-GTGTCTGTCCTGGCCCCC-3′

### 2.10. Statistical analysis

All statistical analyses were conducted using R-version 4.1.3. The significance was defined as p-value being less than 0.05 (*p < 0.05, **p < 0.01, and ***p < 0.001).

## 3. Results

**Figure 1.**
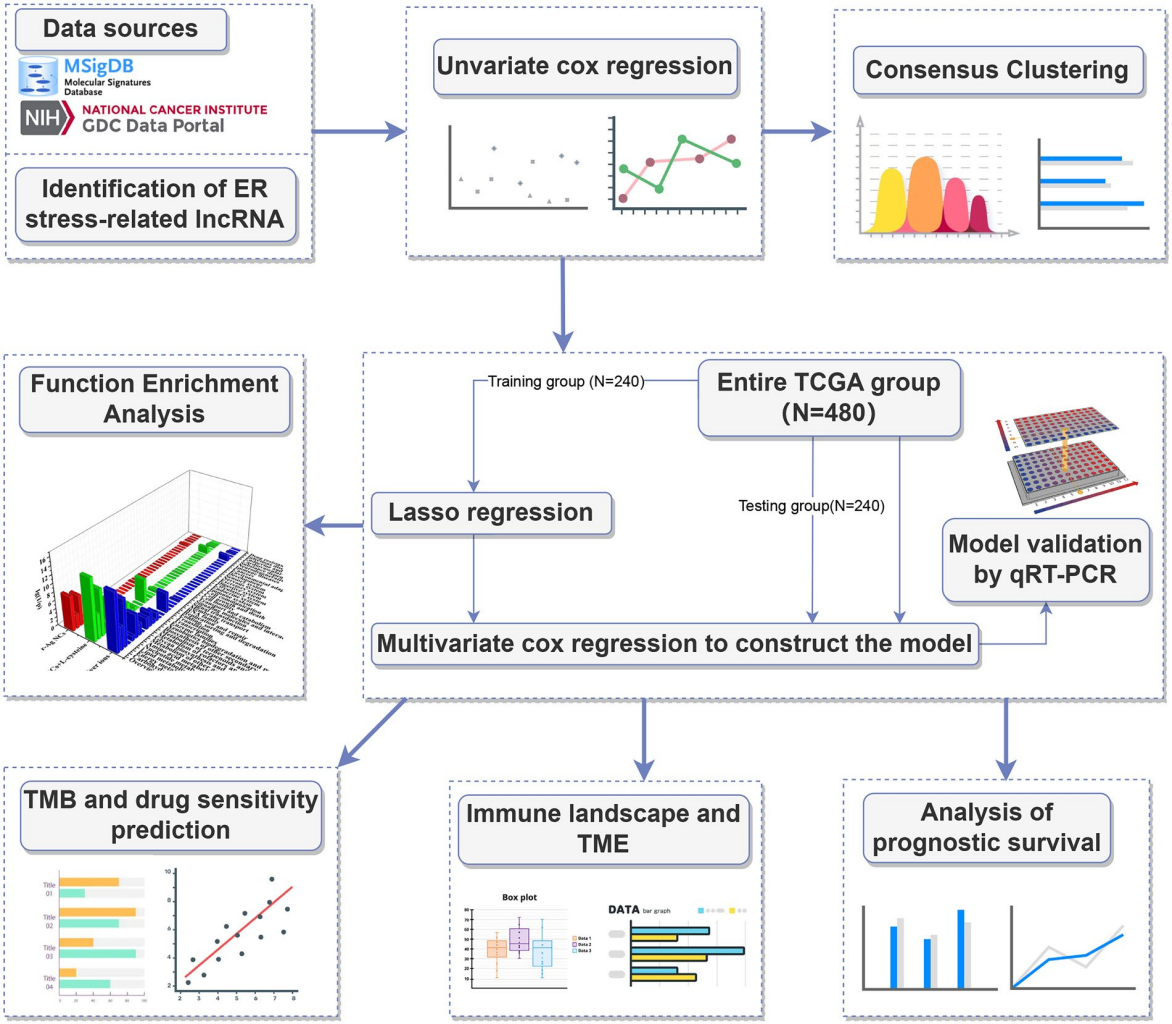
Flowchart of this study.

The study was conducted according to the flow chart shown in Figure 1.

### 3.1. Identification of ERs-related lncRNA signature

**Figure 2.**
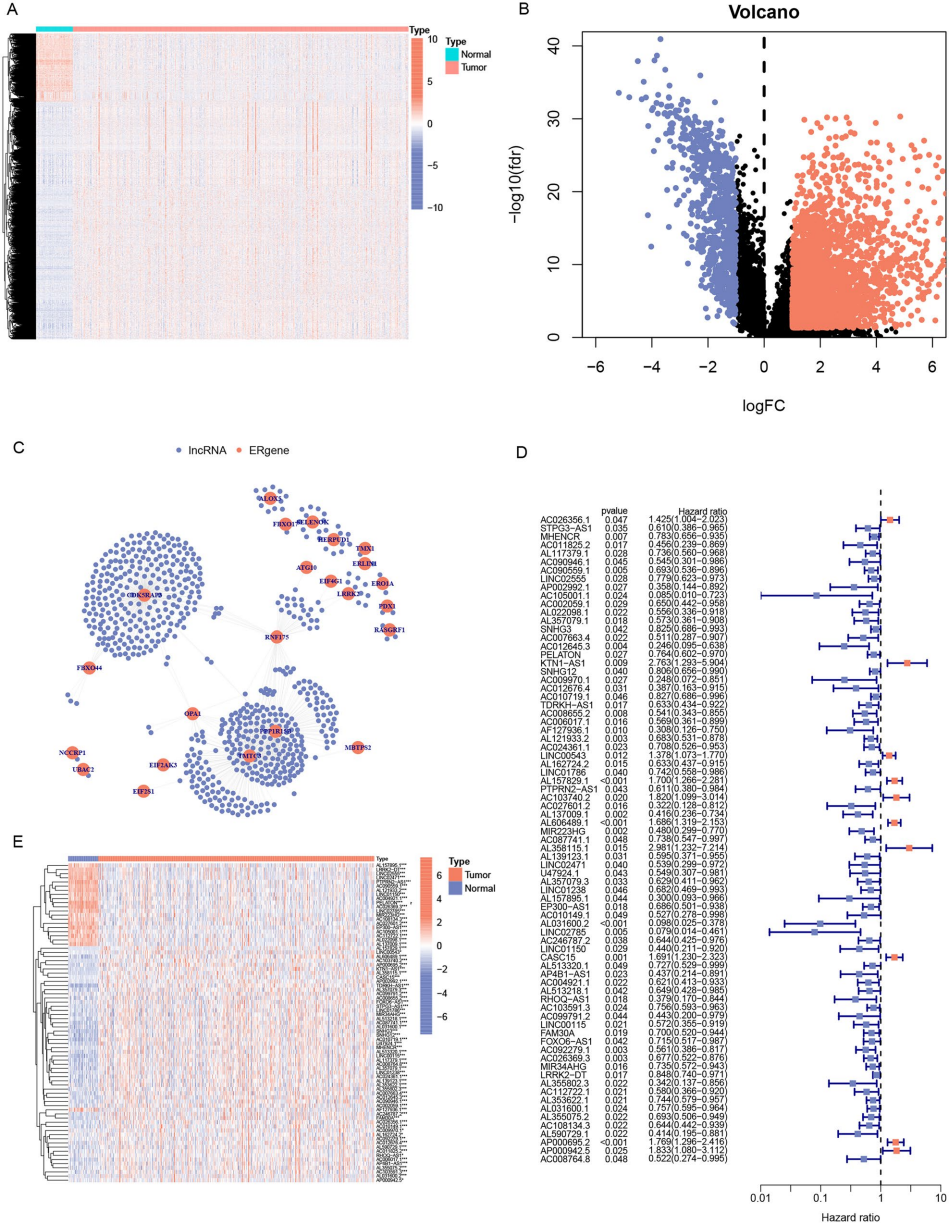
Screening prognosis-related ERs-related lncRNAs as modelling candidates. Heatmap (A) and a volcano plot (B) of differentially expressed lncRNAs in LUAD. (C) The interaction network diagram between ERs-related genes and ERs-related lncRNAs. (D) 77 ERs-related lncRNAs related to prognosis obtained by univariate Cox regression analysis. (E) Differential expression Heatmap of 77 prognosis-related ERs-related lncRNAs. *, **, and *** represent *p* < 0.05, *p* < 0.01, and *p* < 0.001, respectively.

To identify ER stress-related genes, seven groups of ER stress-related genes were downloaded. After removing the overlapping genes, 256 ER stress-related genes were obtained (Table S1). Then a heatmap was plotted to illustrate the expression of ER stress-related genes (Supplementary Figure 1(A)) and 41 potential ER stress-related genes associated with prognostic were identified by univariate Cox hazard regression (Supplementary Figure 1(B)). We downloaded the lncRNA expression profile from the TCGA database and screened 3435 lncRNAs differentially expressed in cancer and normal tissues of LUAD (Figure 2(A, B), Table S2). Then Pearson’s correlation analysis (|Pearson R| > 0.4 and *p* < 0.001) was utilized between 3435 differential lncRNAs and 41 potential prognostic ER stress-related genes, 639 ERs-related lncRNAs were identified at last (Table S3), and Figure 2(C) shows the network diagram of ERs-related lncRNAs.

**Figure 3.**
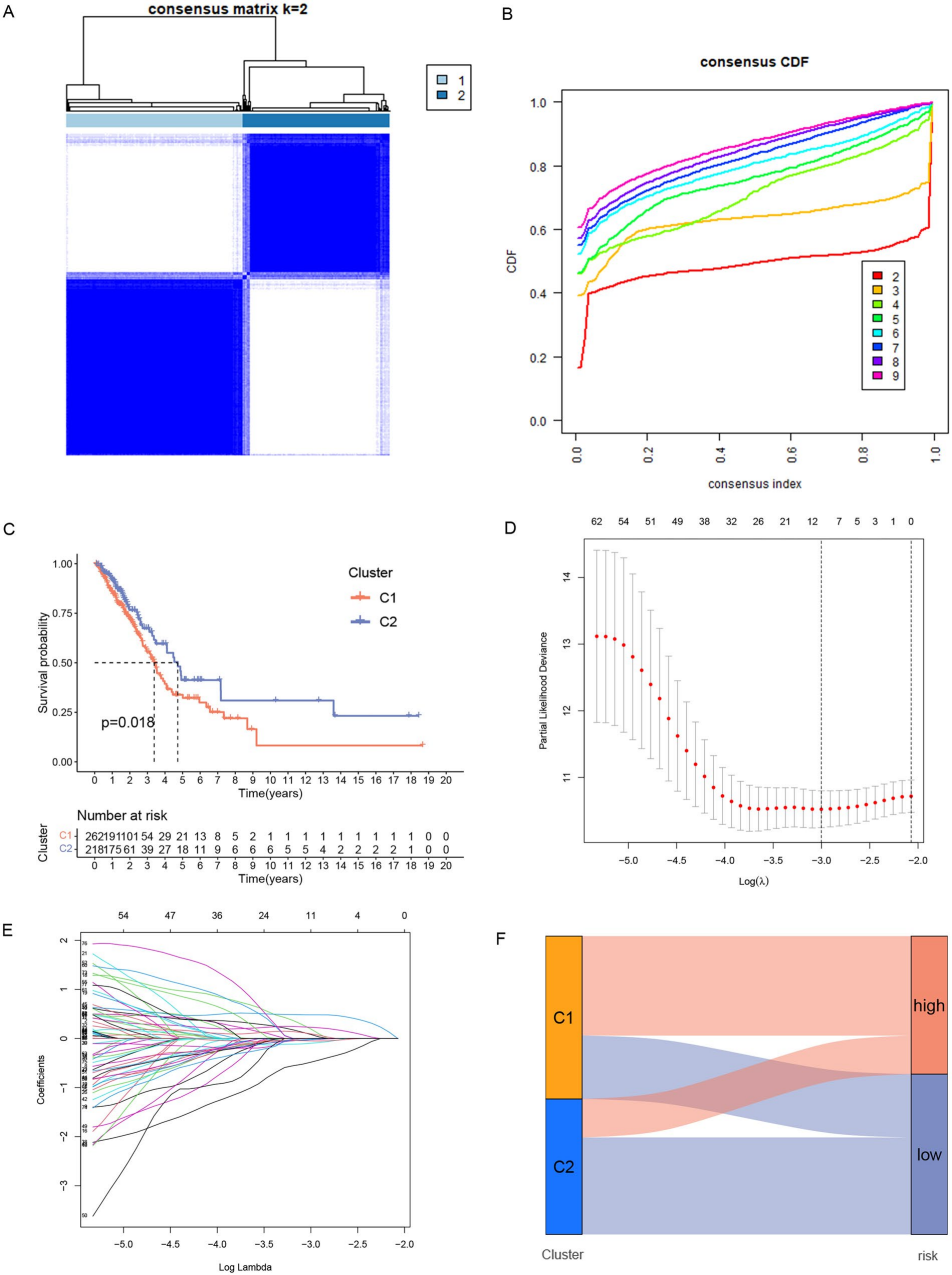
Identification of ERs-related lncRNA Signature. (A) Consensus clustering grouping based on prognosis-related ERs-related LncSig. (B) Empirical cumulative distribution function (CDF) plots display consensus distributions for each k. (C) Survival analysis of two cluster grouping. (D) LASSO coefficient profiles of the 77 prognostic-related ERs-related lncRNAs in the training set. (E) Cross-validation for optimal parameter selection in the LASSO regression. (F) A Sankey diagram of the distribution of samples grouped by cluster and model. CDF, cumulative distribution function.

77 significantly prognostic-related ERs-related lncRNAs were identified by Univariate Cox regression analysis as candidate ERs-related lncRNAs for constructing the prognosis prediction model (Figure 2(D, E)). Based on the expression profile of the 77 ERs-related lncRNAs, the cases can be divided into two clusters clearly by the consensus clustering analysis: Cluster 1 (*n* = 262) and Cluster 2 (*n* = 218) (Figure 3(A, B)). We used Kaplan-Meier analysis to explore the difference between the two groups in prognosis, and the results showed that the prognosis of Cluster 2 was better, while Cluster 1 was worse (Figure 3(C)). Next, all TCGA-LUAD cases were randomly classified into two sets, training sets, and testing sets, at the ratio of 1:1. We used the training set to construct the prognosis prediction model, and the testing set and the entire TCGA set were used to validate the established model. We performed LASSO analysis of 77 ERs-related lncRNAs in the training set to further screen for prognostic factors (Figure 3(D, E)), and then after multivariate Cox proportional hazards regression analysis, we screened out 5 ERs-related lncRNAs for the construction of prediction signature (Table 1). Based on the risk coefficients, we designed a risk score formula for LUAD patients’ survival prediction. The risk score was calculated as follows:

**Table 1.** Multivariate Cox regression analysis of five prognostic lncRNAs.

Gene	coef	HR	HR.95L	HR.95H	*p*-value
AC026356.1	0.621167	1.861099	1.102734	3.141002	0.020009
′KTN1-AS1′	1.09201	2.98026	0.782372	11.35259	0.109532
AL606489.1	0.324128	1.382824	0.933072	2.049361	0.106346
MIR223HG	−1.49848	0.22347	0.093689	0.533028	0.000729
CASC15	0.736351	2.088302	1.316225	3.313269	0.001768

HR*:* hazard ratio; coef: risk coefficients.

Risk score = (0.621) × expression quantity of AC026356.1 + (1.092) × expression quantity of KTN1-AS1+ (0.324) × expression quantity of AL606489.1 + (-1.498) × expression quantity of MIR223HG + (0.736) × expression quantity of CASC15.

In this formula, the positive coefficient tends to be a detrimental factor for the survival of patients, and conversely, the negative coefficient is often a favourable factor for the survival of patients. Cases were divided into a high-risk group and a low-risk group by the median risk score, and the Sankey diagram showed that risk grouping and clustering grouping were consistent in predicting the prognosis of LUAD patients. The samples of the high-risk group with poor prognosis are mostly distributed in Cluster1 with poor prognosis, while the samples of the low-risk group with a good prognosis are mostly distributed in Cluster2 with good prognosis (Figure 3F).

### 3.2. Validation of ERs-related lncRNA Signature

To verify the reliability of the prediction signature, we next analyzed the prediction effect of the signature. In the training set, testing set, and the entire TCGA set, cases were divided into high- and low-risk groups by the median risk score, and Table S4 presented the clinical features of these three sets.

**Figure 4.**
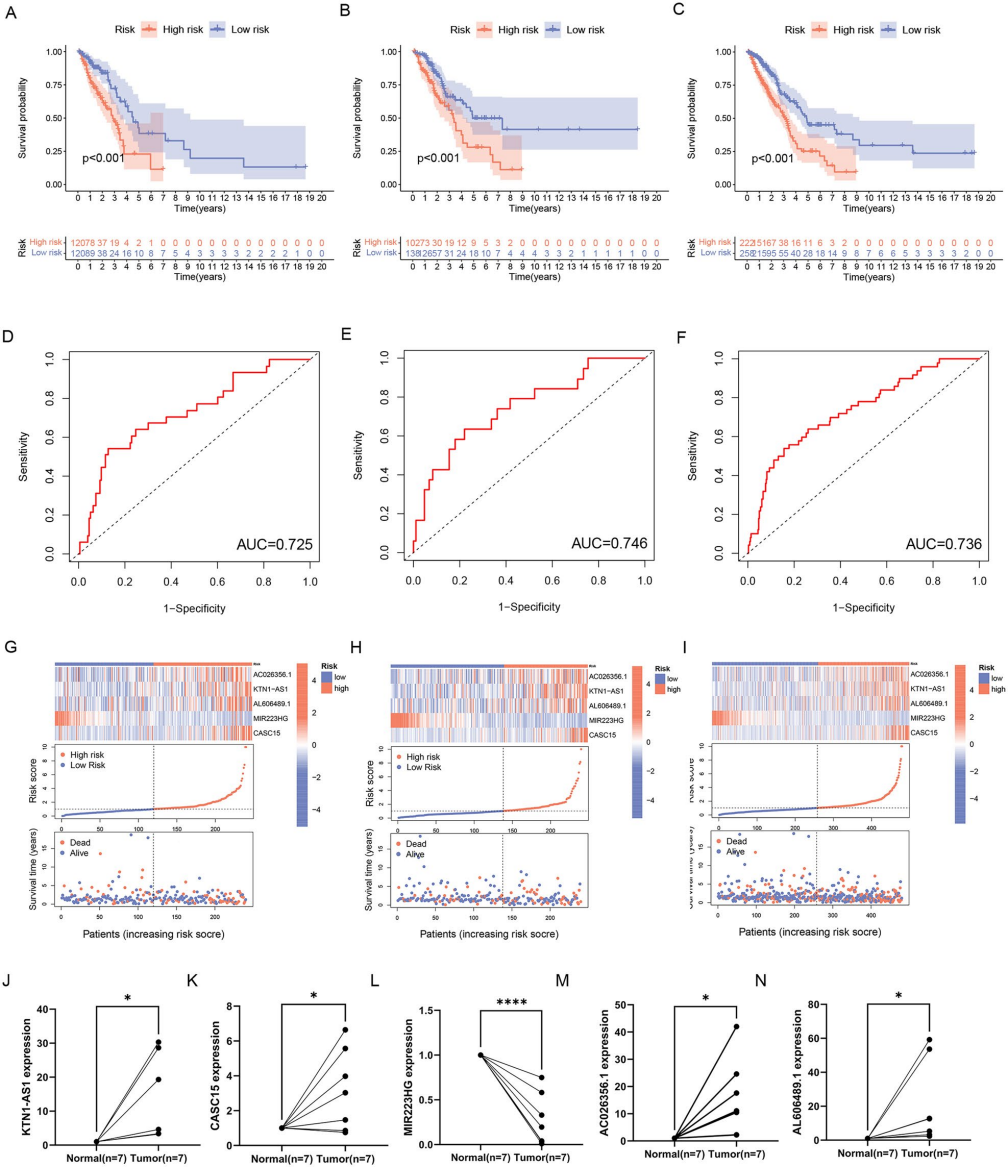
Validation of ERs-related LncSig for survival prediction. (A-C) Different Kaplan-Meier curves in the two risk groups stratified by ERs-related LncSig in the training set (A), testing set (B), and entire TCGA set (C). (D-F) ROC curves of the ERs-related LncSig at 1 year in the training set (D), testing set (E), and entire TCGA set (F). (G-I) In training set (G), testing set (H), and entire TCGA set (I), the expression of ERs-related LncSig in each patient, the distribution of risk grade, and the survival status between the two risk groups. (J-N) Differential expression of five model lncRNAs in tumour tissues and normal tissues. ROC, receiver operating characteristic; AUC, the area under the curve; *, **, and *** represent *p* < 0.05, *p* < 0.01, and *p* < 0.001, respectively.

First, we used the PCA to analyze the distribution of the two risk groups. based on the expression profile of total genes, 256 ERs-related genes, 639 ERs-related lncRNAs, and ERs-related LncSig, respectively (Supplementary Figure 2(A-D)). The results showed that ERs-related LncSig had better grouping ability compared with the other groupings. Next, we analyzed the survival of the different risk groups by Kaplan-Meier analysis and used ROC curves to verify the prediction accuracy. The Kaplan-Meier plot showed that there are all significant differences in the survival curves between high-risk and low-risk groups in these three sets (Figure 4(A-C)), and the AUC values of ROC curves of the three sets were greater than 0.7, indicating that ERs-related LncSig had quite an accurate prediction performance for the prognosis (Figure 4(D-F)). In the three sets, the expression of ERs-related LncSig in each patient, the distribution of risk grade, and the survival status between the two risk groups were shown in Figure 4(G-1).

Then, we validated the differential expression of the five risk lncRNAs included in ERs-related LncSig by qRT-PCR analysis based on clinically obtained paired LUAD tissues from humans. In our samples, the expression of KTN1-AS1, CASC15, AC026356.1 and AL606489.1 was higher in tumour tissues than that in paired normal tissues. On the contrary, MIR223HG was remarkably downregulated in LUAD tissues (Figure 4(J–N)). In addition, the differential expression trend of model genes in TCGA data was consistent with the trend in tissues (Supplementary Figure 3(A-E)), which demonstrates the accuracy of our analysis.

Moreover, we also analyzed the relationship between the expression of each model gene and OS and found that the expression of five model lncRNAs was significantly correlated with the survival of LUAD (Supplementary Figure 3(F-J)).

### 3.3. Relationship between ERs-related LncSig and clinical features

**Figure 5.**
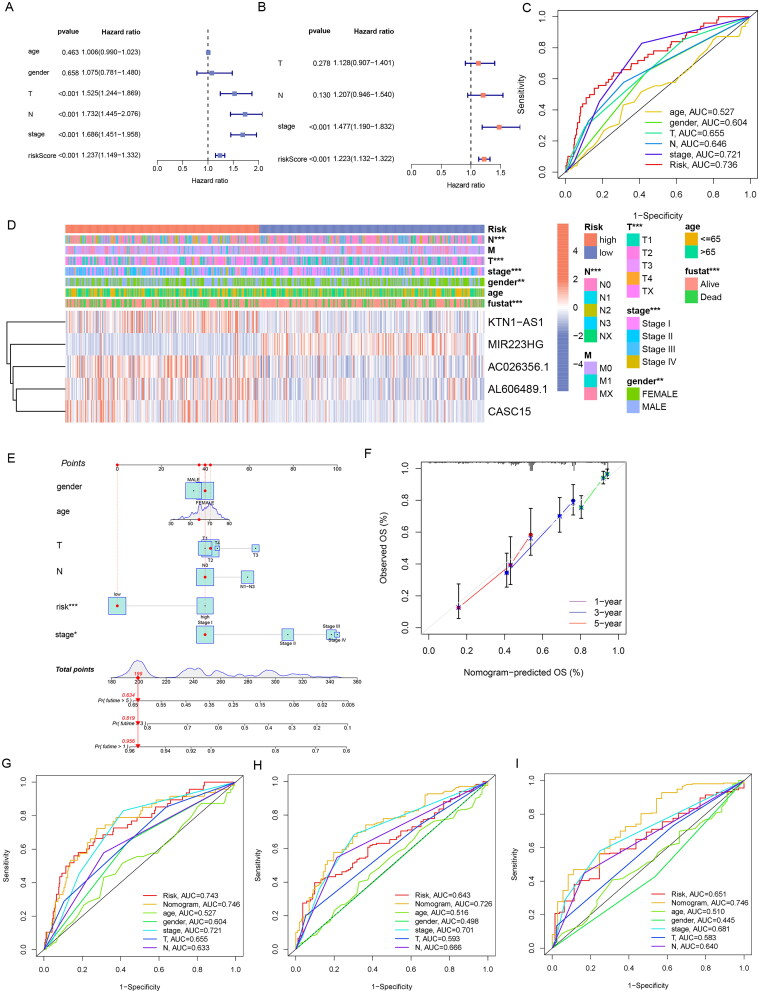
Correlation validation of risk model with clinical characteristics. (A) In the entire TCGA set, univariate independent prognostic analysis of ERs-related LncSig. (B) Multivariate independent prognostic analysis of ERs-related LncSig. (C) Comparison of the 1‑year ROC curve of the ERs-related LncSig and the ROC curves of other clinicopathological features in the entire TCGA set. (D) Heatmap for ERs-related LncSig and the correlation between clinical features and the risk groups. (E) A nomogram predicting 1-, 3-, and 5-year OS of LUAD patients. (F) The calibration curves of the nomogram for 1-year, 3-year, and 5-year survival in LUAD patients. (G-I) ROC curves for prognostic indicators at 1,3 and 5 years. *, **, and *** represent *p* < 0.05, *p* < 0.01, and *p* < 0.001, respectively.

We conducted univariate and multivariate Cox regression analyses to explore whether ERs-related LncSig is an independent prognostic factor in LUAD (Figure 5(A, B)). The ROC curve showed the accuracy of these clinical traits in predicting prognosis (Figure 5(C)).

We found that the predictive performance of ERs-related LncSig was higher than that of other clinical traits in the entire TCGA set. The independent prognostic analysis results and the ROC curve results were also equally excellent in the analysis of the training and testing sets (Supplementary Figure 4(A-F)), and ERs-related LncSig was strongly associated with T, N, and stage as well as survival status (*p* < 0.001) (Figure 5(D)). Meanwhile, the results of stratification analysis detected that the prognostic value of ERs-related LncSig in different subgroups was significant (Supplementary Figure 5).

A nomogram based on risk signature and clinical features was constructed to quantify the 1-, 3-, and 5-year survival probability (Figure 5(E)). The calibration graph is used to analyze the accuracy of the nomogram (Figure 5(F)).

We analyzed the AUC values of the clinical prognostic nomogram at 1, 3, and 5 years, and the values were 0.746, 0.726, and 0.746, respectively (Figure 5(G-I)). Our nomogram had better predictive accuracy than pathological stage and risk score alone.

### 3.4. Functional analysis

**Figure 6.**
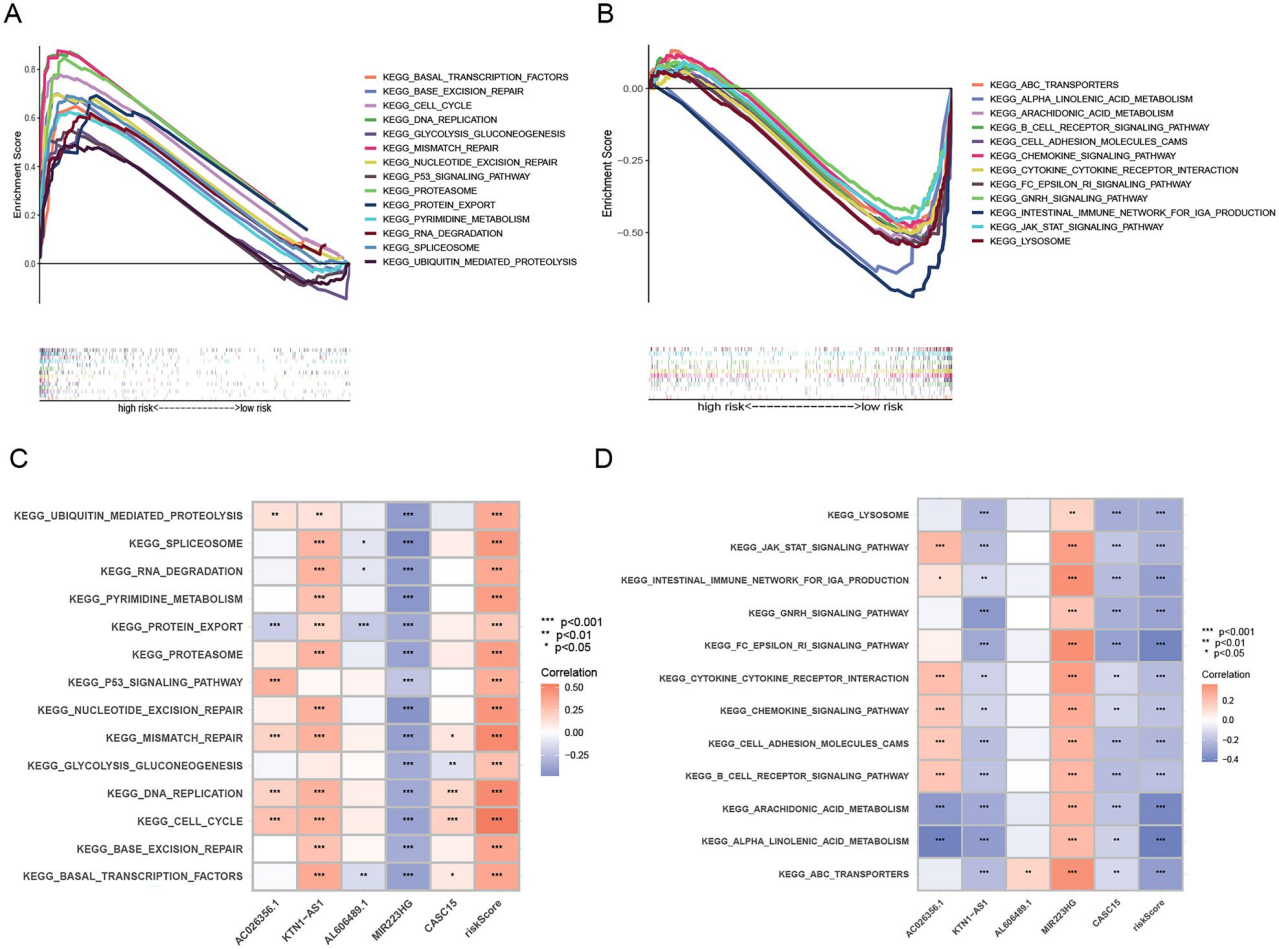
GSEA analysis of ERs-related lncRNA Signature. (A) GSEA analysis of high-risk group calculated by ERs-related LncSig. (B) GSEA analysis of low-risk group calculated by ERs-related LncSig. (C) Correlation of GSEA enriched pathways with each model gene in the high-risk group. (D) Correlation of GSEA enriched pathways with each model gene in the low-risk group.

To determine the potential mechanisms and pathways behind high-risk groups with poor prognosis, we performed GSEA enrichment analysis and explored the correlation of model genes with enrichment results by GSVA analysis. The results showed that 26 pathways were significantly enriched in the high-risk group, and 23 pathways were significantly enriched in the low-risk group (FDR < 0.05) (Figure 6(A, B), Table S5, 6). The pathways enriched in the high-risk group not only included “cell cycle”, the classical cancer-related pathway “p53 signalling pathway”, but also some pathways related to DNA repair and some pathways related to protein degradation, and these enriched results may be related to the UPR and endoplasmic reticulum-associated degradation (ERAD) by ER stress. Among the pathways enriched in the low-risk group, the immune-related pathways such as “Fc epsilon RI signalling pathway”, “Intestinal immune network for IgA production” and “B cell receptor signalling pathway” might explain why the low-risk group had a better prognosis (Figure 6(C, D)).

**Figure 7.**
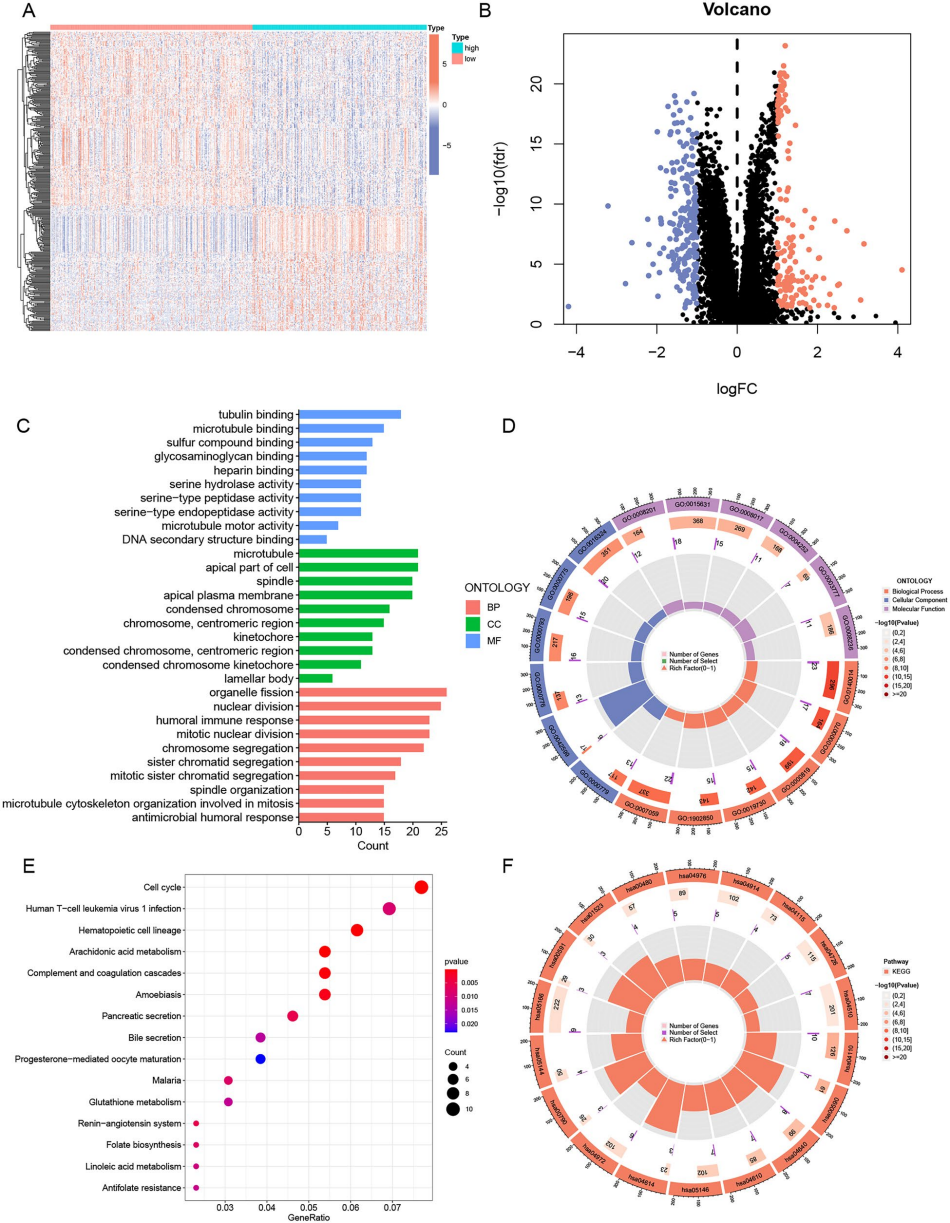
GO and KEGG analysis of ERs-related lncRNA Signature. (A) A Heatmap of DEGs for high- and low-risk groups. (B) A volcano plot of DEGs for high- and low-risk groups. (C-D) Bar chart and circle diagram of the most highly significant enriched results of GO analysis by risk group. (E-F) Bubble plot and circle diagram of the most highly significant enriched results of KEGG analysis by risk group. BP, biological process; CC, cellular component; MF, molecular function.

To analyse the potential biological functions of differentially expressed genes (DEGs) in the two risk groups, we next performed GO and KEGG analyses of DEGs (Figure 7(A, B), Table S7). We found that these differential genes were mainly associated with mitotic biological processes (Figure 7(C, D)), such as “mitotic nuclear division” and “chromosome segregation”. It suggested that the high-risk subgroup worked more vigorously in terms of cell proliferation than the low-risk subgroup. In addition, “humoral immune response” may indicate that the prognostic difference resulting from ERs-related LncSig is immune-related. In the KEGG pathway analysis, the immune-related pathway “Complement and coagulation cascades” was found, and we also obtained some consistent results with the GSEA analysis, such as cell cycle and arachidonic acid metabolism (Figure 7(E, F)).

We concluded from the above analysis that significant molecular functional differences between high and low-risk groups, which provided useful information to speculate the underlying mechanism of ERs-related LncSig in mediating LUAD progression.

### 3.5. Immune and TME analysis

**Figure 8.**
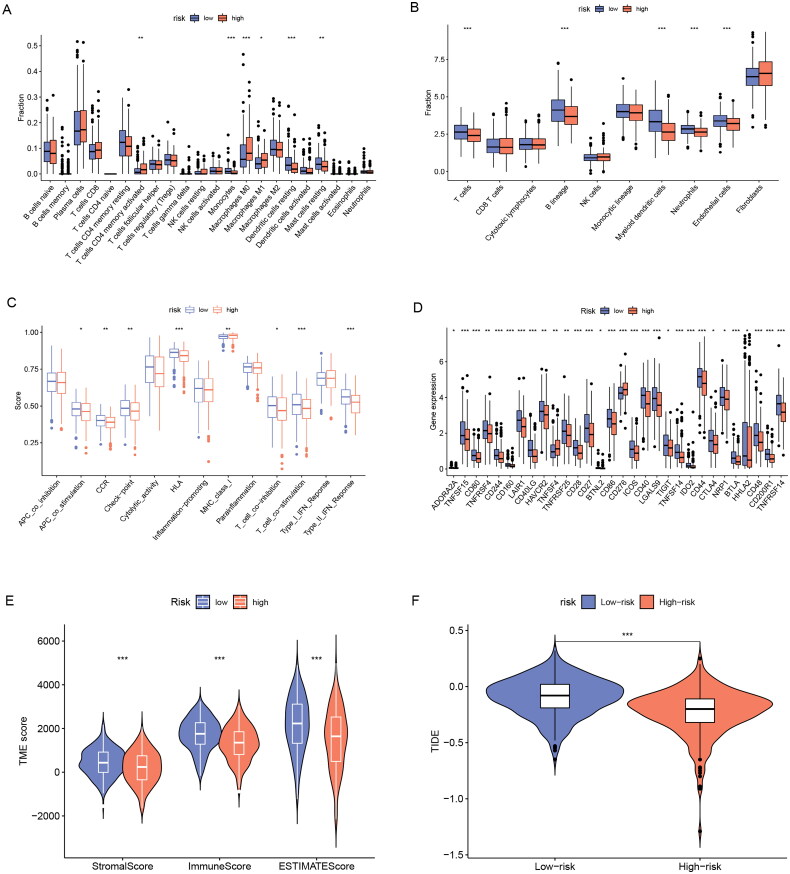
Immune and TME analysis of ERs-related lncRNA Signature. (A) The analysis of tumour immune cell infiltration in high-risk and low-risk groups using the CIBERSORT algorithm. (B) The analysis of tumour immune cell infiltration in high-risk and low-risk groups using MCPcounter algorithm. (C) Differences in immune function between high-risk and low-risk groups. (D) Immune checkpoint differences between high-risk and low-risk groups. (E) The differences in stromal score, immune score, and ESTIMATE score between the low- and high-risk groups. (F) TIDE differences between high-risk and low-risk groups. APC, antigen-presenting cell; CCR, chemokine receptor; HLA, human leukocyte antigen; MHC, major histocompatibility complex; IFN, interferon; *, **, and *** represent *p* < 0.05, *p* < 0.01, and *p* < 0.001, respectively.

It has been shown that ER stress can impair the protective function of innate immune cells in the tumour microenvironment and thus promote tumour progression[15]. Here, we analyzed the relationship between tumour immunity and the tumour microenvironment with ERs-related LncSig. First, we analyzed the differences in TIICs between high- and low-risk groups using the “cibersort” algorithm and the “MCPcounter” algorithm, respectively. The results of the “cibersort” algorithm analysis showed that activated CD4 memory T cells, macrophages M0, and macrophages M1 were increased in the high-risk group; Monocytes, resting Dendritic cells, and resting Mast cells were increased in the low-risk group (Figure 8(A)). Significantly, the Fc epsilon RI pathway in mast cells was already concluded in the previous enrichment results of the GSEA low-risk group. The “MCPcounter” algorithm analysis showed that T cells, B lineage, Myeloid dendritic cells, Neutrophils, and Endothelial cells were decreased in the high-risk group (Figure 8(B)). We also showed the correlation of ERs-related LncSig with immune cell infiltration in other databases (Supplementary Figure 6). We next analyzed the association of ERs-related LncSig with immune function. There were significant differences in immune function scores including CCR (Cytokine-cytokine receptor), Check-point, HLA, MHC class I, T cell co-stimulation, and Type II IFN Response (Figure 8(C)).

Then we analyzed the association of risk grouping with immune checkpoints and showed that 30 immune checkpoint genes were significantly associated with risk grouping (Figure 8(D)). In these immune checkpoint analysis results, we discovered a hot immunotherapeutic molecule, cytotoxic T-lymphocyte-associated protein 4 (CTLA4), which may guide the treatment of CTLA4 antibodies such as ipilimumab in lung adenocarcinoma.

We next evaluated the relationship between risk groupings and the tumour microenvironment. The tumour microenvironment includes an immune cell-dominated immune microenvironment and a fibroblast dominated stromal microenvironment, which underlies tumour growth, migration, and invasion. Interactions among stromal cells, immune cells, and tumour cells are important factors in tumour growth and progression[16]. We found that patients in the low-risk group had significantly higher immune scores, stromal scores, and estimated scores than those in the high-risk group (*p* < 0.001) (Figure 8(E)). We analyzed the association of those three scores with survival in LUAD and found that the lower the three scores, the worse the prognosis (Supplementary Figure 7).

Finally, we evaluated the immunotherapy response by the TIDE algorithm. TIDE is a computational framework developed to evaluate the potential of tumour immune escape from the gene expression profiles of cancer samples. The higher the tide score, the greater the potential of tumour immune escape, indicating that patients were less likely to benefit from immunotherapy. The results indicated that the tide score in the high-risk group was lower than that in the low-risk group, suggesting that patients in the high-risk group benefit more from immunotherapy (Figure 8(F)).

### 3.6. TMB analysis

**Figure 9.**
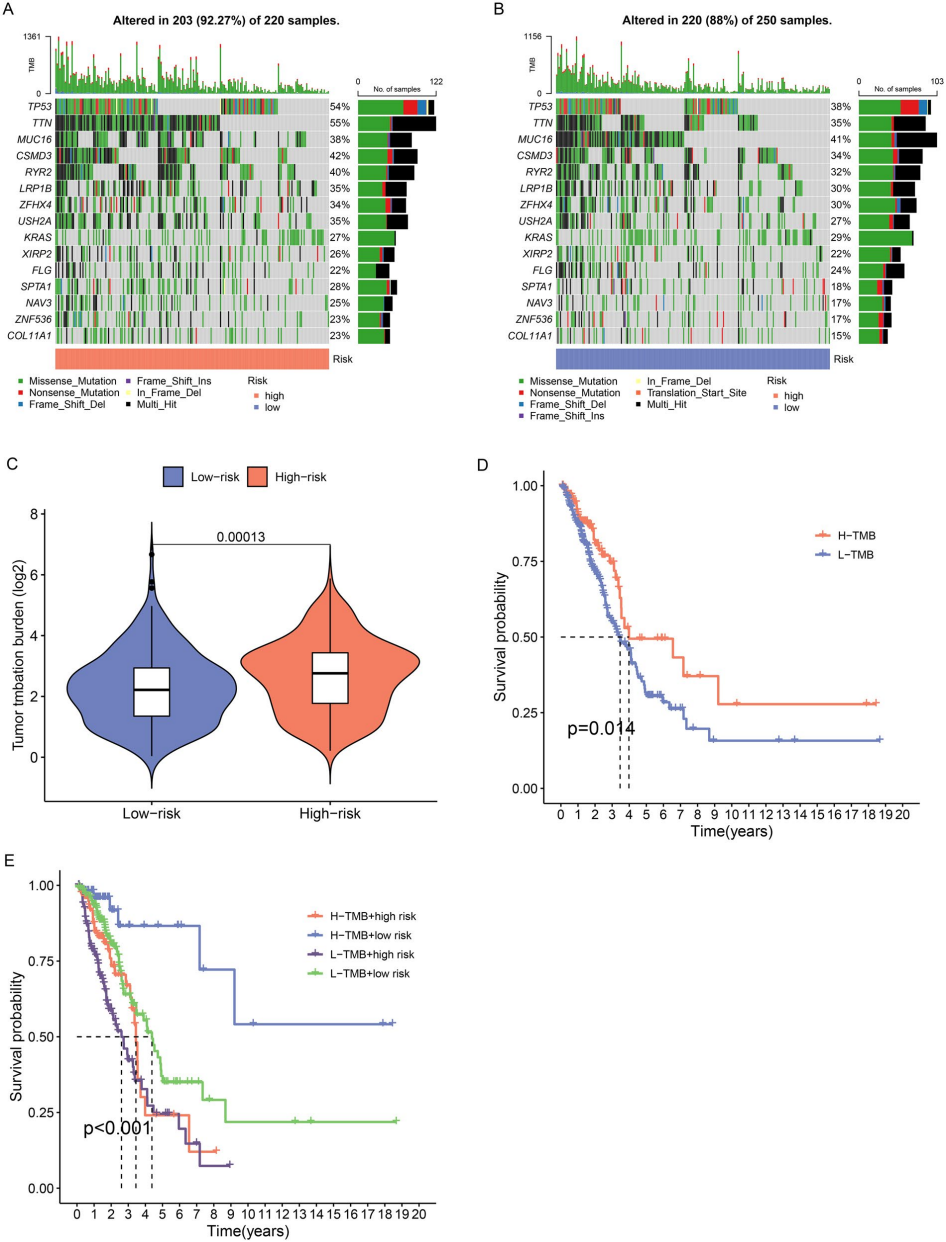
TMB analysis of ERs-related lncRNA Signature. (A) The top 15 genes with the highest mutation rate were in the high-risk group. (B) The top 15 genes with the highest mutation rate were in the low-risk group. (C) TMB differences between high-risk and low-risk groups. (D) Kaplan-Meier survival analysis of high and low TMB patients. **E** Kaplan-Meier analysis of risk groups combined with TMB.

TMB is generally defined as the total number of somatic coding mutations, which is associated with the generation of neoantigens that induce antitumour immunity[17]. Higher TMB scores are generally associated with durable clinical benefit and improved objective response in tumour immunotherapy, and it can predict immunotherapy efficacy in multiple tumours including NSCLC[18, 19]. We calculated the tumour mutation burden of LUAD patients and the result showed the top 15 genes with the highest mutation frequency (Figure 9(A, B)). We found that the high-risk group had significantly higher TMB scores than the low-risk group (*p* < 0.001) (Figure 9(C)). Further survival analysis indicated that patients with high TMB had a better prognosis, and the low-risk subgroup further increased the survival advantage of the high TMB group (Figure 9(D, E)).

### 3.7. Potential drug screening

**Figure 10.**
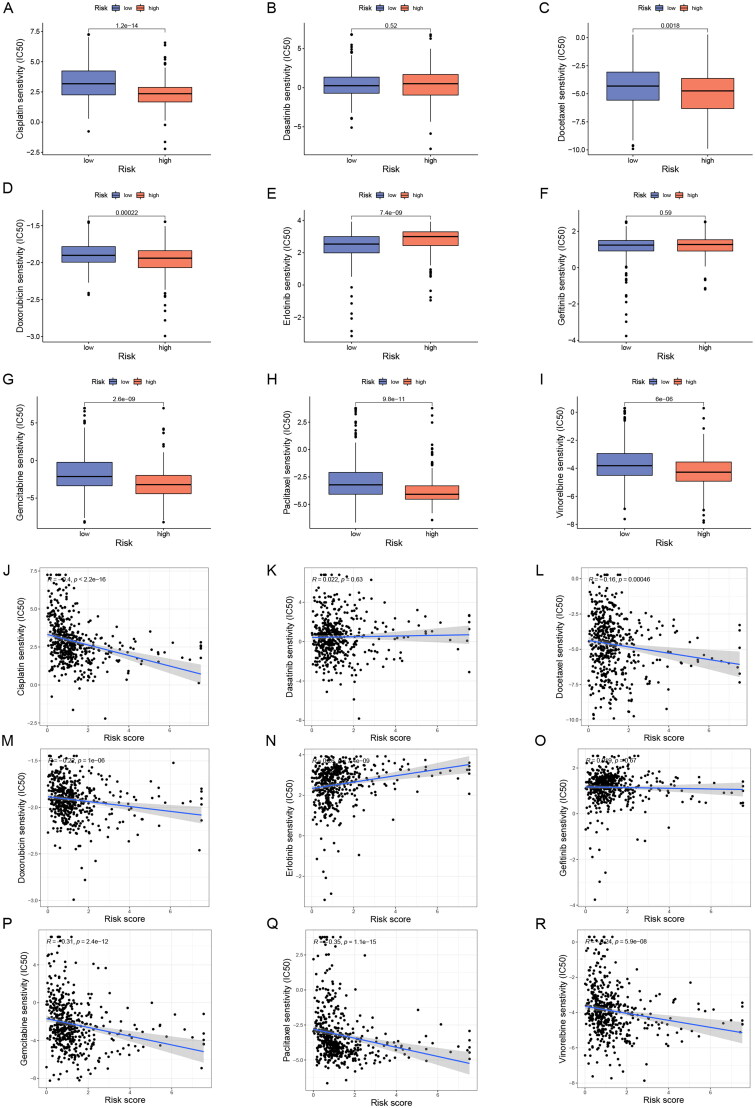
Sensitivity analysis of clinically common chemotherapeutic agents in LUAD. (A-I) The sensitivity difference of commonly used chemotherapeutic agents in LUAD between high- and low-risk groups. (J-R) Correlation plots of risk scores with IC50 of chemotherapeutic agents in TCGA-LUAD patients. IC50, half-maximal inhibitory concentration.

Despite the rapid development of chemotherapeutic drug classes for LUAD, variable sensitivity to drugs in different patients results in unsatisfactory therapeutic outcomes, so a reliable classification criterion is important for the choice of treatment options. To evaluate the predictive effect of ERs-related LncSig on LUAD pharmacotherapy, we analyzed the half-maximal inhibitory concentration (IC50) of commonly used chemotherapeutic agents between high-risk and low-risk groups to evaluate the drug sensitivity of different subgroups. The Wilcoxon test showed that six of these drugs (gemcitabine, paclitaxel, cisplatin, vinorelbine, docetaxel, and doxorubicin) had higher IC50 in low-risk patients (Figure 10(A-R)), suggesting that patients belonged to low-risk are more sensitive to these drugs, while high-risk patients are more sensitive to erlotinib.

In addition, the Connectivity map database provided drug treatment data on two LUAD cell lines, A549 and HCC515, we analyzed the potential compounds associated with ERs-related LncSig in LUAD cell lines based on the differential genes of risk grouping [20]. The top twenty positively correlated and top twenty negatively correlated potential compounds in A549 and HCC515 cell lines are displayed in Table S8, S9, with a larger absolute value of the score representing a stronger correlation of the compound with ERs-related LncSig. Patients in the high-risk group may benefit from compounds with negative correlation and patients in the low-risk group may benefit from compounds with positive correlation.

## 4. Discussion

Despite the progress made in treatment, the overall survival rate of LUAD is still unsatisfactory due to the lack of reliable early prognostic indicators, and different patients respond differently to treatment because of interindividual variability among patients, which necessitates a reliable prognostic marker for individualized prediction and treatment.

ER stress is an emerging hallmark of cancer. The aberrant metabolism of cancer cells triggers ER stress and activates the UPR of the ER. High levels of UPR provide survival advantages for cancer cells[2]. Several studies have demonstrated that the ER stress response pathway plays an important role in cancer initiation and progression, and the therapeutic efficacy of cancer can be improved by modulating the ER stress process. For example, Icariside II can partially enhance cisplatin-induced apoptosis by promoting ER stress signalling in NSCLC[21]. There are also some researches suggesting that Chalcomoracin can increase the sensitivity of lung cancer cells to radiotherapy by enhancing endoplasmic reticulum stress[22]. Notably, many studies have proved that lncRNAs and ER stress can regulate each other and jointly affect tumour progression, but studies on the prognostic biomarkers of LUAD and the tumour-promoting mechanisms associated with ERs-related lncRNAs are still lacking. Therefore, we identified ERs-related LncSig affecting LUAD prognosis and comprehensively analyzed the relationship of ERs-related LncSig with survival prognosis, functional enrichment, TMB, immunity, and TME. We found in the functional analysis that the high-risk group was closely related to cell proliferation and DNA repair, while the better prognosis of the low-risk group might be related to immunity. The results of TIICs analysis showed that ERs-related LncSig was associated with some tumour-infiltrating immune cells. For infiltrating immune cells with significant differences (*p* < 0.001), most immune cells except macrophages showed decreased infiltration in the high-risk group. Immune checkpoint analysis revealed that ERs-related LncSig is closely associated with multiple immune checkpoints, and the majority of immune checkpoint genes showed reduced expression in the high-risk group. All these differences in high- and low-risk groups may lead to different prognoses and serve as promising targets for immunotherapy. We analyzed the relationship between risk grouping and the tumor microenvironment and found that our risk grouping is closely related to the tumour microenvironment. Then we applied the TIDE score to assess the response of risk subgroups to immunotherapy and found that patients in the high-risk group may benefit more from immunotherapy.

TMB is an emerging biomarker that predicts response to immunotherapy. In tumour immunotherapy, higher TMB scores are generally associated with better immunotherapy response[23]. In our analysis, the TMB score was higher in the high-risk group than in the low-risk group, indicating that patients in the high-risk group might benefit more from immunotherapy, the same as the results of our TIDE analysis. We also performed drug sensitivity prediction to evaluate the predictive effect of ERs-related LncSig on LUAD drug treatment, which will facilitate personalized medicine for patients.

Among the model component genes, CASC15 is generally considered a cancer-promoting factor and has been extensively studied in several cancer types, such as non-small cell lung cancer, melanoma, ovarian cancer and colorectal cancer[24–27]. KTN1-AS1 is also generally regarded as a cancer-promoting factor and plays a cancer-promoting role in non-small cell lung cancer, bladder cancer, hepatocellular carcinoma, ovarian cancer, glioma, and lymphoma[28–33]. However, the roles of another three lncRNAs in tumorigenesis and development have not been specifically investigated, and our study reveals their potential roles in LUAD.

Although we performed the above analysis and got good results, there are still several limitations in this study. Firstly, we preliminarily explored the related functions involved in the regulatory network of different risk groups, but the specific mechanisms underlying their effects on LUAD prognosis still need further investigation. Secondly, although this model was validated in the TCGA dataset, a large number of external datasets are still needed to verify its applicability in clinical patients. Finally, *in vivo* and *in vitro* functional experiments should be performed to further confirm our findings and explore the specific mechanism of action.

## 5. Conclusion

Using ER stress, a unique intracellular biological process, as an entry point, we explored ER stress-related lncRNAs in LUAD, and constructed a clinical survival prognosis model using the expression profiles of these lncRNAs, and explored the model’s relationship with patient survival, immunity, TMB and drug sensitivity. This study may provide new insights into clinical decision-making and individualized medication for lung adenocarcinoma and provide new ideas for basic research.

## Ethical approval

The authors are accountable for all aspects of the work in ensuring that questions related to the accuracy or integrity of any part of the work are appropriately investigated and resolved. The trial was conducted in accordance with the Declaration of Helsinki (as revised in 2013). The use of human tissue research was approved by the Institutional Research Ethics Committee of the Harbin Medical University Cancer Hospital. Informed consent was obtained from all patients and written informed consent was signed.

## Supplementary Material

Supplemental Material

## Data Availability

All data will be provided upon request to the corresponding author.
