# Supplementary material for: Identification of endoplasmic reticulum stress-related lncRNAs in lung adenocarcinoma by bioinformatics and experimental validation
Source: Ann Med. 2023 Aug 29;55(2):2251500. doi: 10.1080/07853890.2023.2251500 (PMC10467521; doi:10.1080/07853890.2023.2251500)
Supplement: Supplemental Material [file IANN_A_2251500_SM2154.zip › suppl_data/Supplementary documents for flow charts and detailed methods.docx]

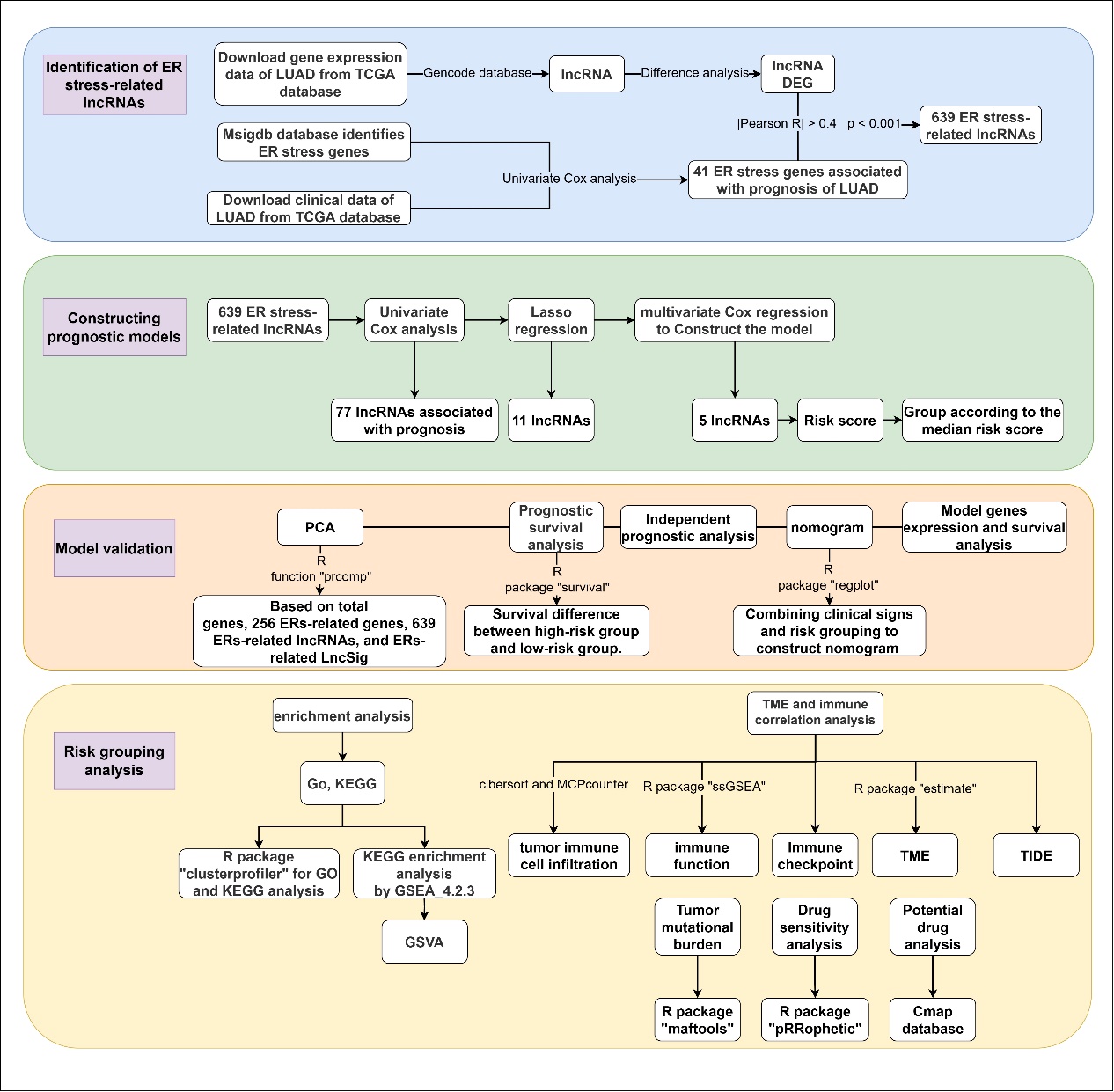


**Detailed flow chart**

**Identification of ER stress-related lncRNAs**

The ER stress-related gene set was downloaded from the molecular signature database, including the following gene sets:

GOBP_NEGATIVE_REGULATION_OF_RESPONSE_TO_ENDOPLASMIC_RETICULUM_STRESS; GOBP_POSITIVE_REGULATION_OF_RESPONSE_TO_ENDOPLASMIC_RETICULUM_STRESS; GOBP_POSITIVE_REGULATION_OF_TRANSLATION_IN_RESPONSE_TO_ENDOPLASMIC_RETICULUM_STRESS; GOBP_REGULATION_OF_RESPONSE_TO_ENDOPLASMIC_RETICULUM_STRESS; GOBP_REGULATION_OF_TRANSLATION_IN_RESPONSE_TO_ENDOPLASMIC_RETICULUM_STRESS; GOBP_REGULATION_OF_TRANSLATION_INITIATION_IN_RESPONSE_TO_ENDOPLASMIC_RETICULUM_STRESS; GOBP_RESPONSE_TO_ENDOPLASMIC_RETICULUM_STRESS.

Duplicate genes were manually removed. Then, Download transcriptome data and clinical data of TCGA lung adenocarcinoma.

These ER stress-related gene expression profile data were combined with the survival time and survival status of clinical cases, and then univariate Cox regression analysis was performed using the function “coxph” in the R package “survival” to screen the ER stress genes associated with the prognosis of lung adenocarcinoma. All univariate Cox regression analyses in the article used this function.

On the other hand, lncRNAs were screened out in TCGA LUAD gene expression profile, and differentially expressed lncRNAs were screened out with R package “limma” (logFC>1, FDR<0.05).

The differentially expressed lncRNAs and ER stress-related genes were analyzed by R function “cor.test” (|Pearson R| > 0.4 and p < 0.001), and the ER stress-related lncRNAs were obtained.

**Constructing prognostic models**

Combined with clinical data, 77 ER stress-related lncRNAs with potential prognostic value were identified by univariate Cox regression analysis (pvalue<0.05).

Then, the R package “glmnet” was used for LASSO analysis to further screen variables, optimize candidate genes for modeling, find the position with the smallest cross validation error, and find the optimal log(λ). Finally, five ER stress-related lncRNAs were screened out by multivariate Cox regression analysis to construct the prediction model. The TCGA cases were divided into high and low risk groups according to the median score of the risk score.

**Model validation**

R function "prcomp" was used to conduct PCA analysis based on total genes, 256 ERs-related genes, 639 ERs-related lncRNAs, and ERs-related LncSig respectively to verify the rationality of grouping. Then, combined with the clinical data, the R package "survival" was used to verify the prognostic difference between the two risk groups. An independent prognostic analysis of the risk score was then performed to demonstrate that the risk score could be used as an independent prognostic factor. Nomogram was constructed using R package "regplot" combined with clinical signs and risk grouping. The ROC diagrams in this paper were made with R package " timeROC ".

**Risk grouping analysis**

According to the differential genes of high-risk and low-risk groups, GO and KEGG enrichment analysis was performed through the R package “clusterProfiler”. Then GSEA enrichment analysis software GSEA_4.2.3 was used for KEGG enrichment analysis, and then the relationship between each model gene and related pathways was analyzed with R package “GSVA”.

Tumor microenvironment and immune correlation analysis are mainly divided into five aspects: The relationship between risk grouping and tumor immune cell infection was verified by cibersort and MCPcounter algorithms; The relationship between immune function and risk grouping was achieved by ssGSEA analysis; The relationship between risk grouping and immune checkpoint gene expression is reflected by the differential expression of immune checkpoints in high-risk and low-risk subgroups; For tumor microenvironment analysis, first obtain the tumor microenvironment score file with the R package “estimate”, and then calculate the difference of each score in high-risk and low-risk groups; For TIDE analysis, first upload the expression file to TIDE website (<http://tide.dfci.harvard.edu/>) for scoring, and then analyze the difference of TIDE score between high-risk and low-risk groups.

For the analysis of tumor mutational burden, tumor mutation data were downloaded in TCGA, and the R package “maftools” was applied to visualize the mutated genes in high-risk and low-risk groups. At the same time, the relationship between mutation burden and survival was compared between the two risk groups.

In terms of drug sensitivity, the R package “pRRophetic” was used to predict the relationship between different risk groups and commonly used drug sensitivity, and the correlation was calculated by Spearman method. For the prediction of potential therapeutic drugs, we first analyze the differential genes between high-risk and low-risk groups (p<0.05), and input the high and low expressed genes into CMAP database (https://clue.io/) to calculate the potential compounds.
