# Supplementary material for: Identification of endoplasmic reticulum stress-related lncRNAs in lung adenocarcinoma by bioinformatics and experimental validation
Source: Ann Med. 2023 Aug 29;55(2):2251500. doi: 10.1080/07853890.2023.2251500 (PMC10467521; doi:10.1080/07853890.2023.2251500)
Supplement: Supplemental Material [file IANN_A_2251500_SM2154.zip › suppl_data/Supplementary Information.docx]

**Supplementary Information**

**Identification of endoplasmic reticulum stress-related lncRNAs in lung adenocarcinoma by bioinformatics and experimental validation**

Tong Xin et al.

**Supplementary Tables**

**Table S1.** After removing the overlapping genes, 256 ER stress-related genes were obtained finally.

**Table S2.** The differentially expressed lncRNAs in LUAD and normal tissues based on TCGA database.

**Table S3.** Endoplasmic reticulum stress-related lncRNAs obtained by Pearson correlation analysis (|Pearson R| > 0.4 and P < 0.001).

**Table S4.** Clinical features of three LUAD patients sets.

**Table S5.** GSEA Pathways enriched in high-risk group

**Table S6.** GSEA Pathways enriched in low-risk group

**Table S7.** The differentially expressed genes in high- and low-risk groups based on TCGA database.

**Table S8.** The top twenty positively correlated potential compounds in A549 and HCC515 cell lines analyzed by Cmap database.

**Table S9.** The top twenty negatively correlated potential compounds in A549 and HCC515 cell lines analyzed by Cmap database.

**
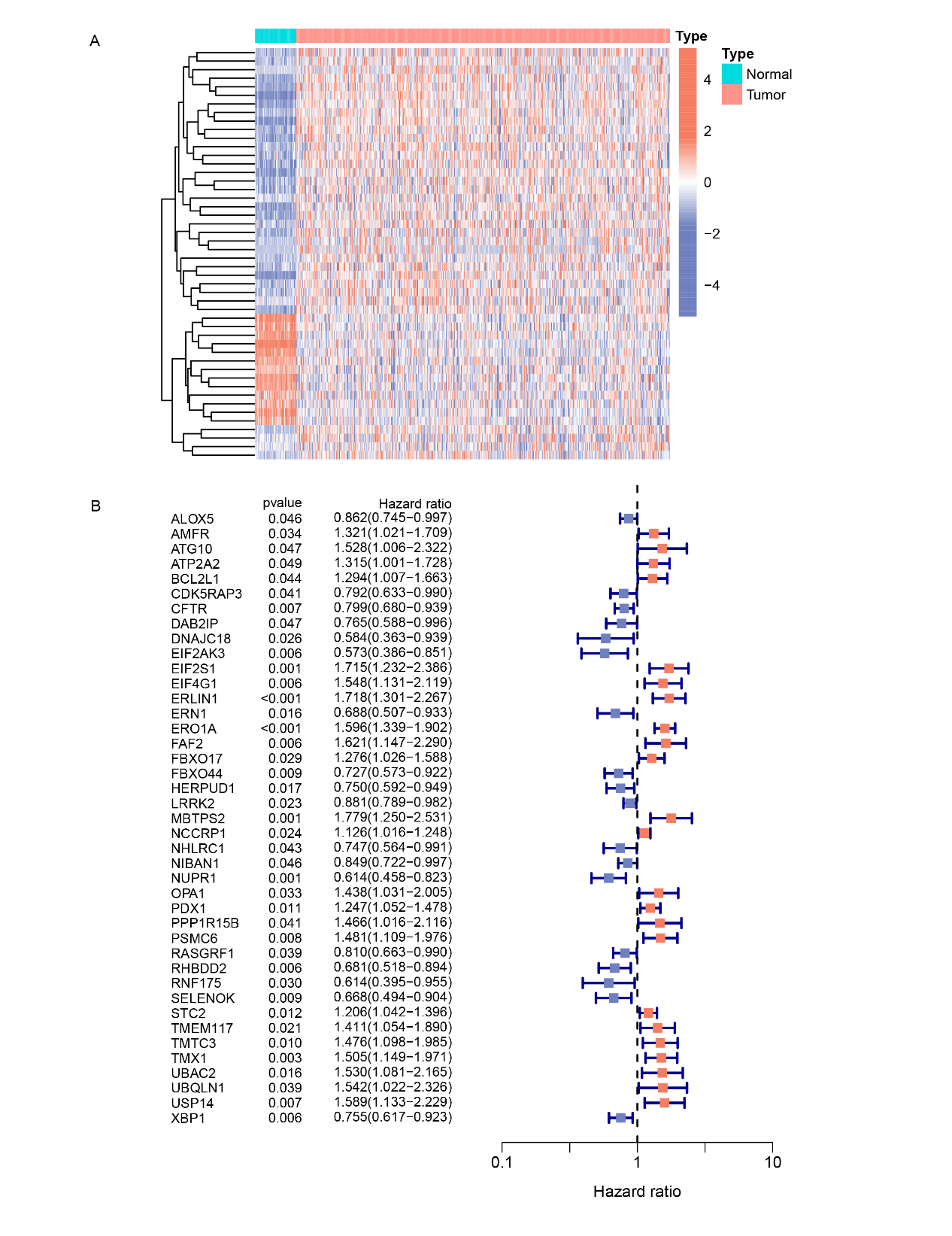
**

**Supplementary Fig. 1.** Screening of prognostically relevant ER stress genes. (A) Heatmap of the expression of 256 ERs-related genes in LUAD. (B) ERs-related genes associated with survival from univariate Cox analysis.


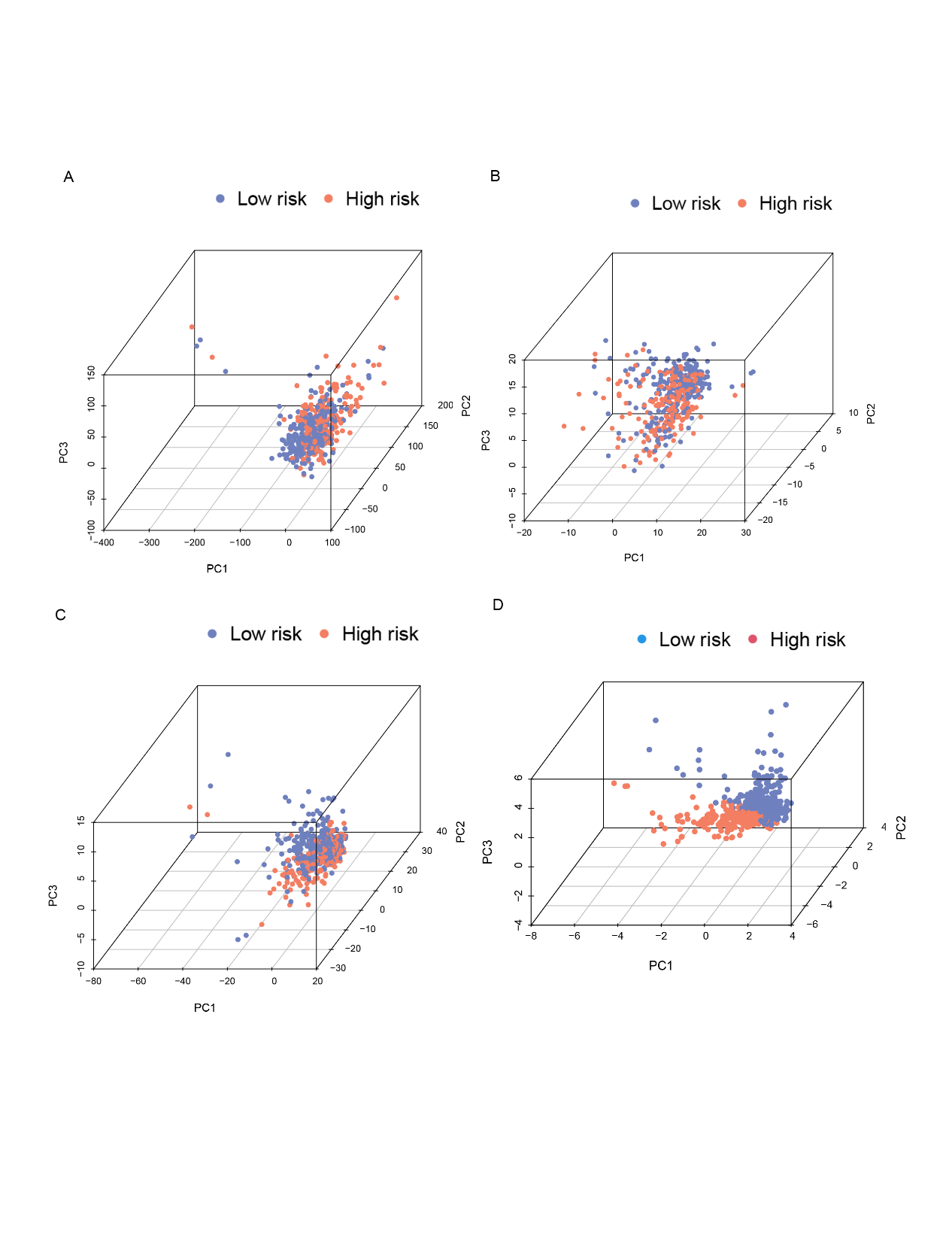


**Supplementary Fig. 2.** PCA is based on the expression profile of (A) total genes, (B) 256 ERs-related genes, (C) 639 ERs-related lncRNAs (D), and ERs-related LncSig.


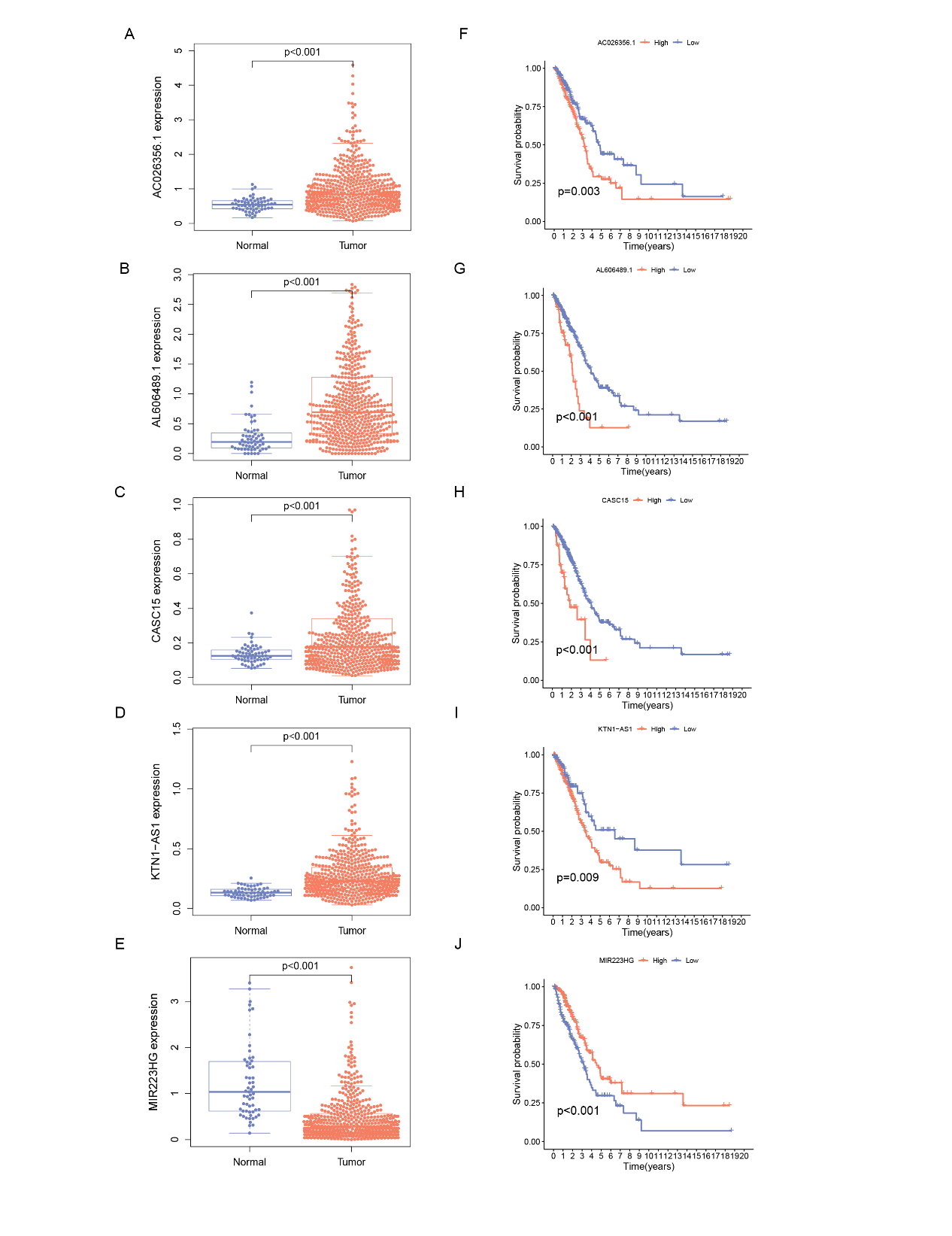


**Supplementary Fig. 3.** (A-E) The scatterplot of 5 differentially expressed model lncRNAs in LUAD and normal samples. (F-J) The Kaplan-Meier analysis is based on the expression of 5 model lncRNAs.


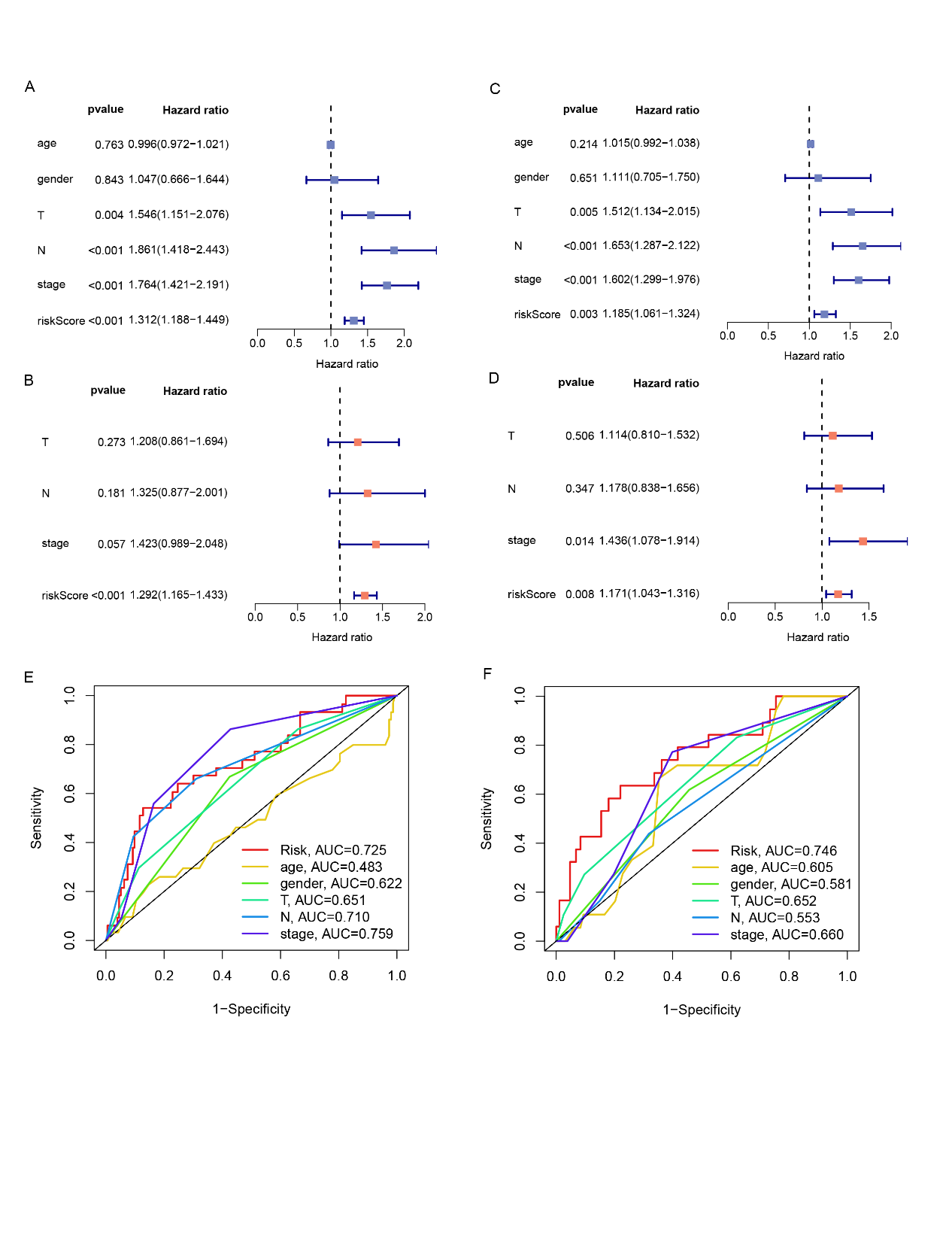


**Supplementary Fig. 4.** Univariate and multivariate independent prognostic analysis in training (A-B) and testing (C-D) sets. Comparison of the 1‑year ROC curve of the ERs-related LncSig and the ROC curves of other clinicopathological features in the training set (E) and testing set (F).


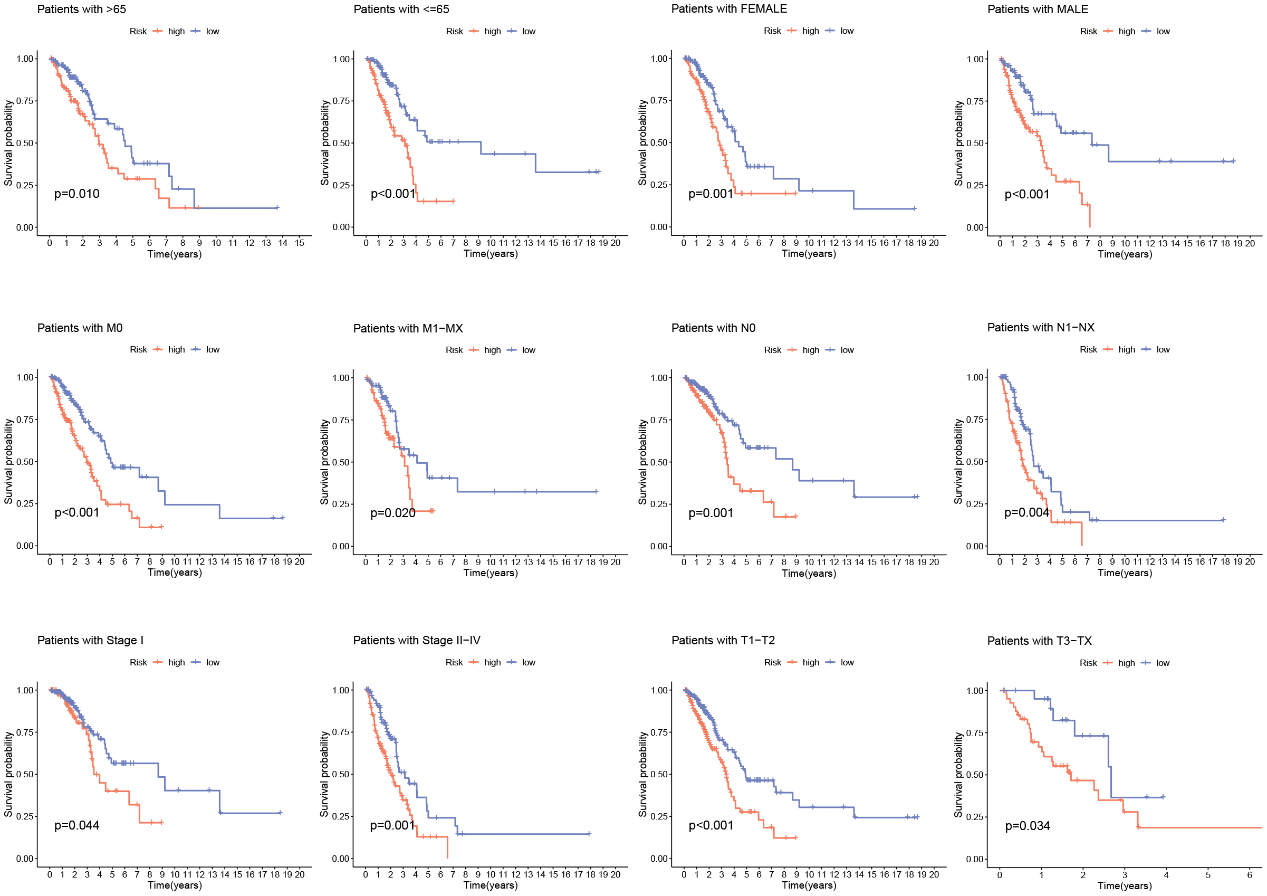


**Supplementary Fig. 5.** Survival curves of high- and low-risk groups grouped in different clinical characteristics subgroups.


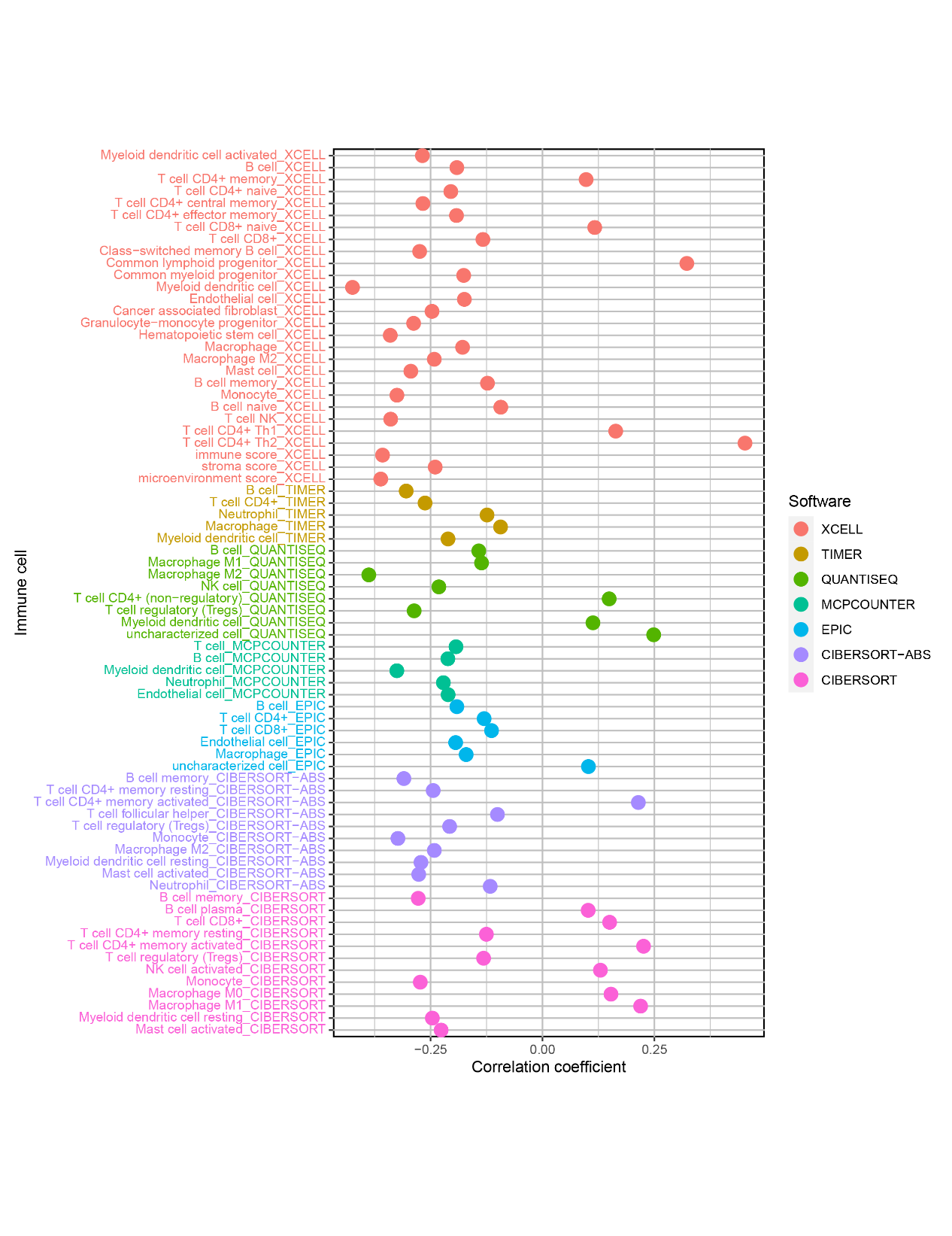


**Supplementary Fig.** **6.** TIICs analysis of risk groups in different databases.


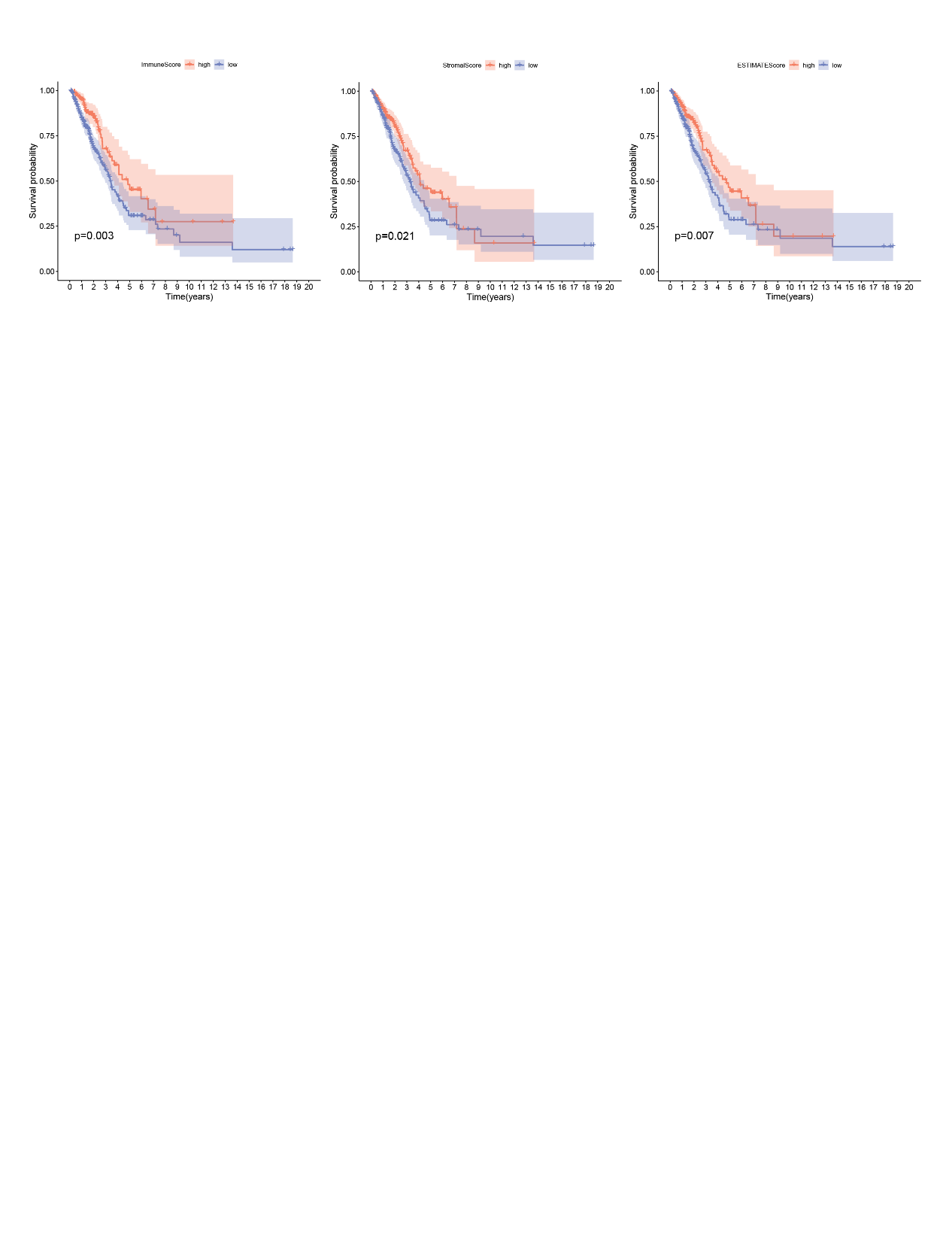


**Supplementary Fig. 7.** Association of the immune score, stromal score, and estimated score with patient survival in LUAD.
